# Amniotic Fluid Mesenchymal Stromal Cells Derived from Fetuses with Isolated Cardiac Defects Exhibit Decreased Proliferation and Cardiomyogenic Potential

**DOI:** 10.3390/biology12040552

**Published:** 2023-04-05

**Authors:** Manali Jain, Neeta Singh, Raunaq Fatima, Aditya Nachanekar, Mandakini Pradhan, Soniya Nityanand, Chandra Prakash Chaturvedi

**Affiliations:** 1 Stem Cell Research Center, Department of Hematology, Sanjay Gandhi Post-Graduate Institute of Medical Sciences, Lucknow 226014, India; 2 Department of Maternal Reproductive Health, Sanjay Gandhi Post-Graduate Institute of Medical Sciences, Lucknow 226014, India

**Keywords:** isolated congenital heart defects, amniotic fluid, mesenchymal stromal cells, cardiomyogenic potential, cardiac transcription factors

## Abstract

**Simple Summary:**

Congenital heart defect (CHD) is the most common birth defect that affects the structure of the heart from birth. A possible reason for this occurrence could be alterations in the properties of the stem cells associated with fetal heart development. In this study, we compared the growth and cardiomyogenic potential of fetal-derived amniotic fluid mesenchymal stem cells (AF-MSCs) from isolated congenital heart defective fetuses (ICHD) with AF-MSCs of normal fetuses. The ICHD AF-MSCs showed a defect in the ability to grow efficiently and displayed increased senescence and DNA damage processes compared to normal AF-MSCs. Furthermore, the ICHD AF-MSCs showed cardiomyogenic differentiation defects that were accompanied by decreased expression of various proteins, including cardiac progenitor markers, transcription factors, and structural proteins, which are necessary for proper heart development. Overall, our study highlights that these defects in AF-MSCs of ICHD fetuses possibly contribute to CHDs and may lead to improper heart development.

**Abstract:**

Amniotic fluid mesenchymal stromal cells (AF-MSCs) represent an autologous cell source to ameliorate congenital heart defects (CHDs) in children. The AF-MSCs, having cardiomyogenic potential and being of fetal origin, may reflect the physiological and pathological changes in the fetal heart during embryogenesis. Hence, the study of defects in the functional properties of these stem cells during fetal heart development will help obtain a better understanding of the cause of neonatal CHDs. Therefore, in the present study, we compared the proliferative and cardiomyogenic potential of AF-MSCs derived from ICHD fetuses (ICHD AF-MSCs) with AF-MSCs from structurally normal fetuses (normal AF-MSCs). Compared to normal AF-MSCs, the ICHD AF-MSCs showed comparable immunophenotypic MSC marker expression and adipogenic and chondrogenic differentiation potential, with decreased proliferation, higher senescence, increased expression of DNA-damaged genes, and osteogenic differentiation potential. Furthermore, the expression of cardiac progenitor markers (PDGFR-α, VEGFR-2, and SSEA-1), cardiac transcription factors (GATA-4, NKx 2-5, ISL-1, TBX-5, TBX-18, and MeF-2C), and cardiovascular markers (cTNT, CD31, and α-SMA) were significantly reduced in ICHD AF-MSCs. Overall, these results suggest that the AF-MSCs of ICHD fetuses have proliferation defects with significantly decreased cardiomyogenic differentiation potential. Thus, these defects in ICHD AF-MSCs highlight that the impaired heart development in ICHD fetuses may be due to defects in the stem cells associated with heart development during embryogenesis.

## 1. Introduction

Congenital heart defects (CHDs) affect 1% of stillbirths per annum globally and represent the most prevalent neonatal disorder [1]. CHDs are characterized by mutations in cardiac transcription factors, single nucleotide polymorphism (SNPs), aneuploidy, and chromosomal copy number variants (CNV), which result in diverse phenotypes of CHDs involving conotruncal and aortic arch artery abnormalities, left ventricular outflow tract defects, valvular defects, and cardiac septation defects [2,3,4,5]. These cardiac defects can occur as an isolated CHD independently or with a wide variety of non-cardiac anomalies. Isolated congenital heart defects (ICHDs) are the most fatal subtype of CHDs [6]. A significant proportion of fetuses with ICHDs die in the neonatal period if no treatment is provided [7]. Current treatments, such as fetal cardiac interventions, cardiac catheterization, or heart surgery, allow the patients to survive. Apart from being expensive and available at tertiary centers, there is also the risk of rejection, thrombosis, and inflammation, which limits their use; thus, heart transplantation remains the only choice [8]. Considering the limitations, such as the scarcity of potential donor matches and the limited capacity of the heart to proliferate cardiomyocytes and regenerate the damaged myocardium, there is an urgent need to explore better alternatives for CHDs. Recently, stem cell therapy has emerged as a potential treatment option for CHDs [9]. Several clinical studies on adult patients with congenital heart disease have been conducted [10,11]. However, the advancements in stem cell regenerative therapy for adults are yet able to be applied to children with CHD.

Mesenchymal stromal cells (MSCs) are the most widely explored stem cells in pre-clinical and clinical studies as cellular therapy modules, particularly in cardiac repair and regeneration. MSCs, obtained from both adult and fetal sources such as bone marrow, adipose tissue, umbilical cord blood, and amniotic fluid, have shown cardiomyogenic differentiation potential [12]. However, adult-derived MSCs are limited by invasive harvest processes, low yield, varying self-renewal capacity between cell donors, and a low frequency of MSCs (0.001–0.01%) [13]. On the other hand, different fetal-derived MSCs have also been studied for their potential as an alternative source of cardiac regeneration. MSCs derived from umbilical cord blood, placental tissue, and amnion membrane improved the damaged myocardium, but proliferation and survival were the main concerns [14].

Human amniotic fluid (AF)-derived MSCs also represent a fetal stem cell source that can be easily isolated by prenatal amniocentesis. AF possesses a greater expansion capacity and a higher frequency of MSCs (0.9–1.5%). The AF is the protective liquid present in the amniotic sac and primarily contains amniocytes, amniotic epithelial cells, transcripts, signaling molecules, and numerous metabolites produced by fetal and placental tissue that provide dynamic information about the physiological and pathological conditions in the developing fetus [15]. It has been reported that amniotic epithelial cells contain progenitor cells or resident cardiac stem cells that differentiate into various organs such as the heart, brain, and kidney [16]. These resident cardiac stem cells in AF have been studied for the expression of transcription factors of mesodermal origin, such as *NKx 2-5*, *GATA-4*, and the *TBX* family, which play an essential role in heart development during embryogenesis [17,18]. Thus, any defects in the functional properties of these cardiac stem cells or cardiac progenitor stem cells will contribute to an increased risk of neonatal heart defects as in the case of ICHDs. Our lab has recently demonstrated that the AF-MSCs have better cardiomyogenic potential than bone-marrow-derived MSCs. AF-MSCs derived from structurally normal fetuses exhibit high expression of cardiac progenitor markers, cardiac transcription factors, and cardiovascular markers [19]. We speculate that the AF-MSCs derived from fetuses with isolated congenital heart defects (ICHD AF-MSCs) might possess variations in the proliferation and differentiation potential towards cardiomyogenic lineage. Thus, assessing the alterations in these processes in the ICHD AF-MSCs will provide better insights for understanding the causes of improper heart development in fetuses with ICHD.

Therefore, in the present study, we evaluated the proliferative ability and cardiomyogenic potential of AF-MSCs derived from fetuses with isolated congenital heart defects (ICHD AF-MSCs) and compared them with those of AF-MSCs from normal fetuses (normal AF-MSCs).

## 2. Materials and Methods

### 2.1. Subjects

Seven pregnant women with fetuses having isolated congenital heart defects (ICHDs) and seven pregnant women with structurally normal fetuses were included in the study; the mean age of the participants was 29 years (19–38 years). Written/informed consent was taken from all participants before including them in the study. Inclusion criteria for the cohort of fetuses with ICHD were major congenital heart defects confirmed by fetal echocardiogram, pre-conceptional folic acid intake by mothers, and a similar gestational age period (16–22 weeks). Exclusion criteria included maternal CHD, the presence of a fetal anomaly except cardiac, the mother having gestational diabetes or previous history of diabetes mellitus, multiple gestations, and maternal intake of drugs known to cause congenital heart defects. Women with structurally normal fetuses who underwent routine amniocentesis for prenatal diagnosis and had no cardiac, genetic, or other organ abnormalities were recruited as controls.

### 2.2. Isolation and Culture of Normal AF-MSCs and ICHD AF-MSCs

The isolation and culture of normal AF-MSCs and ICHD AF-MSCs were done as previously described [19]. In brief, after informed consent, approximately 5 mL of AF was collected from pregnant females with normal and ICHD fetuses [number of patients (N) = 7] for each group of gestation age 16–22 weeks during the amniocentesis process for prenatal diagnosis. Amniotic fluid cells were pelleted down at 300× *g* for 5 min and then resuspended in complete minimum essential medium (CMEM), which contains α-minimum essential media (α-MEM), 16.5% fetal bovine serum (FBS), Glutamax (1%), and penicillin-streptomycin (1%). The cells were incubated at 37 °C in a 5% CO_2_ atmosphere for 5 days, and after that, the culture medium was changed with fresh CMEM to remove non-adherent cells. On the 7th day, adherent cells were harvested by trypsinization and expanded to passage 3. Cells from the 3rd passage were used for all the experiments.

### 2.3. Immunophenotypic Characterization of Normal AF-MSCs and ICHD AF-MSCs

Normal AF-MSCs and ICHD AF-MSCs were characterized according to the International Society for Cellular Therapy standard criteria for MSC characterization [20]. The cells were labelled with two-colour fluorophores pre-conjugated monoclonal antibodies: MSC markers, viz. CD73-Phycoerythrin (PE), CD90-Allophycocyanin (APC), CD105-fluorescein isothiocyanate (FITC), and hematopoietic markers: CD34-FITC, CD45-PE, and HLA-DR-APC and were incubated for 45 min in the dark. After washing with 1% BSA in 1 × PBS, the cells were centrifuged at 300× *g* for 5 min and then resuspended in 1 × PBS. All flow-cytometry acquisitions were performed and analyzed on the BD FACS lyric and BD FACSuite, respectively. The concentration of antibodies used in the study is given below in Table 1.

### 2.4. In-Vitro Adipogenic, Osteogenic and Chondrogenic Differentiation

At 70–80% confluency, normal AF-MSCs and ICHD AF-MSCs were induced into adipogenic, osteogenic, and chondrogenic differentiation using the adipogenic, osteogenesis, and chondrogenic differentiation kits (Gibco; Invitrogen, Grand Island, NY, USA) in a 12-well culture plate. The medium was changed twice a week for three weeks. On the 21st day, the cells were fixed, washed, and stained with a working solution of 0.5% Oil Red O (Sigma Aldrich, Saint Louis, MO, USA), 0.2% Alizarin Red (Sigma Aldrich), and 1% Alcian Blue (Sigma-Aldrich) for adipogenic, osteogenic, and chondrogenic differentiation, respectively. The photomicrographs of the stained cells were captured by a Carl Zeiss phase contrast microscope, and the percentage of positively stained cells was quantified by determining the percentage of the stained area (area fraction) to the total area of the cells, using ImageJ.

### 2.5. RNA Isolation and Real-Time PCR

Total RNA was isolated from untreated and tri-lineage differentiated normal and ICHD AF-MSCs using the Trizol reagent (Invitrogen, Waltham, MA, USA). A high-capacity cDNA reverse transcription kit was used to transcribe the mRNA to cDNA (Bio-Rad, Hercules, CA, USA). SYBR Green PCR Master Mix Chemistry was used to analyze the expression of adipogenic gene viz. *PPARγ*, *lipoprotein lipase*, osteogenic genes viz. *RUNX* and *Osteopontin*, chondrogenic gene viz. *SOX9* and *AGCAN* using a quantitative real-time PCR machine (Bio-Rad). The ∆∆Ct method was used to determine the relative fold change expression level for each targeted gene normalized with a housekeeping gene, i.e., *GAPDH*, and fold change expression in the genes was calculated by the 2^−∆∆Ct^ method [21]. The primers used in quantitative real-time PCR are given below in Table 2.

### 2.6. Growth Kinetics

The normal AF-MSCs and ICHDAF-MSCs were plated in triplicate at a concentration of 2 × 10^4^ cells/well in 24-well plates. The cells were harvested and counted every 24 h, up to 192 h. The mean values were used to plot a growth curve, and population doubling time (PDT) was calculated using the following standard formula:

*PDT* = [*log*2/*logN*t – *logN*0] × t

where *N*t is the cell number at a particular culture period, *N*0 is the initial number of cells, and t is the cell culture time in hrs [22].

### 2.7. MTT Assay

The normal AF-MSCs and ICHD AF-MSCs were seeded in 96-well plates at a density of 1 × 10^4^ cells/well for 48 h. After 48 h, 10 µL of MTT solution (Sigma-Aldrich, St. Louis, MO, USA) in PBS (5 mg/mL) was added to each well and incubated in the cell culture incubator for 4 h. The supernatant was removed carefully. The formazan crystals were then dissolved in 100 µL of dimethyl sulfoxide (DMSO, Sigma-Aldrich, St. Louis, MO, USA). Cell viability in each well was determined by optical density measurement at 570 nm.

### 2.8. Senescence-Associated (SA) β-Galactosidase Assay

The Senescence Cells Cytochemical Staining Kit (Sigma-Aldrich Co.) was used to assess SA β-Gal activity. Normal AF-MSCs and ICHD AF-MSCs were plated in 24-well plates in triplicate at a concentration of 2 × 10^4^ cells per well. After 48 h, cells were fixed using a solution of 2% formaldehyde and 0.5% glutaraldehyde for 5 min. Then, cells were washed with PBS followed by incubation at 37 °C for at least 12 h with a staining solution. The assessment of SA β-Gal activity was carried out according to the manufacturer’s instructions, and positive blue staining was used as a biomarker of cellular senescence. The percentage of positively stained cells was estimated by counting at least 100 cells in each sample.

### 2.9. Expression of Senescence and DNA Damage Associated Genes

Total RNA was isolated from both normal and ICHD AF-MSCs using the Trizol reagent (Invitrogen, Waltham, MA, USA). A high-capacity cDNA reverse transcription kit was used to transcribe the mRNA to cDNA (Bio-Rad). SYBR Green PCR Master Mix Chemistry was used to analyze the expression of senescence-associated genes viz. *TP53* and *CDKN1A* and DNA damage-response genes viz. *MRE11*, *NBS1*, and *PARP,* using a quantitative real-time PCR machine (Bio-Rad). The ∆∆Ct method was used to determine the relative fold change expression level for each targeted gene normalized with a housekeeping gene, i.e., *GAPDH*, and fold change expression in the genes was calculated by the 2^−∆∆Ct^ method [21]. The primers used in quantitative real-time PCR are given above in Table 2.

### 2.10. Characterization of Normal AF-MSCs and ICHD AF-MSCs for the Expression of Cardiac Progenitor Markers

Normal AF-MSCs and ICHD AF-MSCs were analyzed for the expression of cardiac progenitor markers, viz. stage-specific embryonic antigen-1 (SSEA-1), vascular endothelial growth factor 2 (VEGFR-2), and platelet-derived growth factor receptor (PDGFRα), by flow cytometry using pre-conjugated antibodies such as PDGFRα-PE, SSEA-1-PE, and VEGFR-2-APC as mentioned above. The dilutions of antibodies used in the study are given in Table 1.

### 2.11. Analysis of Cardiac Transcription Factors Expression in Normal AF-MSCs and ICHD AF-MSCs

Normal AF-MSCs and ICHD AF-MSCs were cultured in CMEM with 10 µM 5′-azacytidine (5′-aza; Sigma-Aldrich MO, USA) to induce cardiomyogenic lineages. After 24 h, the medium was replaced with CMEM without 5′-aza for 21 days, with the medium changing twice a week. Control cells were treated with CMEM alone. The cells were examined for fold changes in the expression of cardiac transcription factors by real-time PCR. RNA was isolated from untreated and 5′-aza-treated normal and ICHD AF-MSCs using the Trizol reagent (Invitrogen, Waltham, MA, USA). A high-capacity cDNA reverse transcription kit was used to transcribe the mRNA to cDNA (Bio-Rad). SYBR Green PCR Master Mix Chemistry was used to analyze the expression of cardiac transcription factors viz. *GATA-4*, *ISL-1*, *NKx 2-5*, *TBX-5*, *TBX-18*, and *MeF-2C* using a quantitative real-time PCR machine (Bio-Rad). The ∆∆Ct method was used to determine the relative fold change expression level for each targeted gene normalized with a housekeeping gene, i.e., *GAPDH,* and fold change expression in the genes was calculated by the 2^−∆∆Ct^ method [21]. The primers used in quantitative real-time PCR are given in Table 2.

### 2.12. Cardiovascular Trilineage Differentiation of Normal AF-MSCs and ICHD AF-MSCs

The cells were cultured in CMEM containing 10 µM of 5′-azacytidine (5′-Aza) to stimulate cardiomyogenic differentiation, and the expression of structural cardiac proteins was detected using the cardiac Troponin T (cTNT) (Abcam) antibody. Untreated and 5′-aza-treated cells were grown at a density of 5 × 10^4^ cells/well in 12-well plates (BD Falcon). Cells were rinsed in 1X PBS after 24 h of incubation and then cultured in CMEM alone for 21 days. On day 21, the cells were fixed with 4% paraformaldehyde (Sigma-Aldrich) and permeabilized for 15 min with 0.5% Triton X-100 in PBS. Blocking was done with 5% goat serum to minimize non-specific binding. The cells were blocked and incubated overnight at 4 °C with the following antibodies: cTNT at a dilution of 1:200. The cells were then washed thrice and incubated with 1:1000 diluted FITC-labeled anti-rabbit secondary antibodies for 1 h at room temperature in the dark. Cells were rinsed thrice with 1X PBS and counterstained with 500 µL of Hoechst dye (Sigma-Aldrich). The acquisition of the stained images was done by a confocal microscope (Zeiss, Peabody, MA, USA), and their analysis was performed by its software, ZEN-blue software (version 2.3, Zeiss Microscopy GmbH, Germany).

### 2.13. Endothelial Differentiation

The cells were treated with CMEM containing 50 ng/mL VEGF and 25 ng/mL BMP4 to induce endothelial differentiation (R&D Systems, Minneapolis, MN, USA). A total of 50,000 cells/well were seeded in triplicate wells of 12-well plates, and the medium was changed every other day for 21 days. The cells were fixed and immunocytochemical assessed for CD31 expression using anti-CD31 antibodies at a dilution of 1:100 according to the protocol described in Section 2.11.

### 2.14. Smooth Muscle Actin Differentiation

To differentiate smooth muscle, the cells were treated with CMEM containing 10 ng/mL TGF-β and 5 ng/mL BMP-4 (R&D Systems). Another 50,000 cells/well were seeded in triplicate wells of 12-well plates and incubated for 24 h at 37 °C in 5% CO_2_ for three weeks. On day 21, cells were fixed, and immunocytochemistry was performed using an antiα-SMA antibody at 1:50 using the protocol described in Section 2.11. The dilution of the antibodies used for the ICC is given in Table 1.

### 2.15. Statistical Data Analysis

Statistical data were analyzed using GraphPad Prism 8 (GraphPad Software, San Diego, CA, USA), and analysis was performed using the student’s t-test. ImageJ and Zen Blue Lite were used to analyze the images. All data are expressed as the mean ± standard deviation (SD).

## 3. Results

### 3.1. Morphology, Immunophenotypic Characterization, and Tri-Lineage Differentiation of Normal AF-MSCs and ICHD AF-MSCs

**Figure 1:**
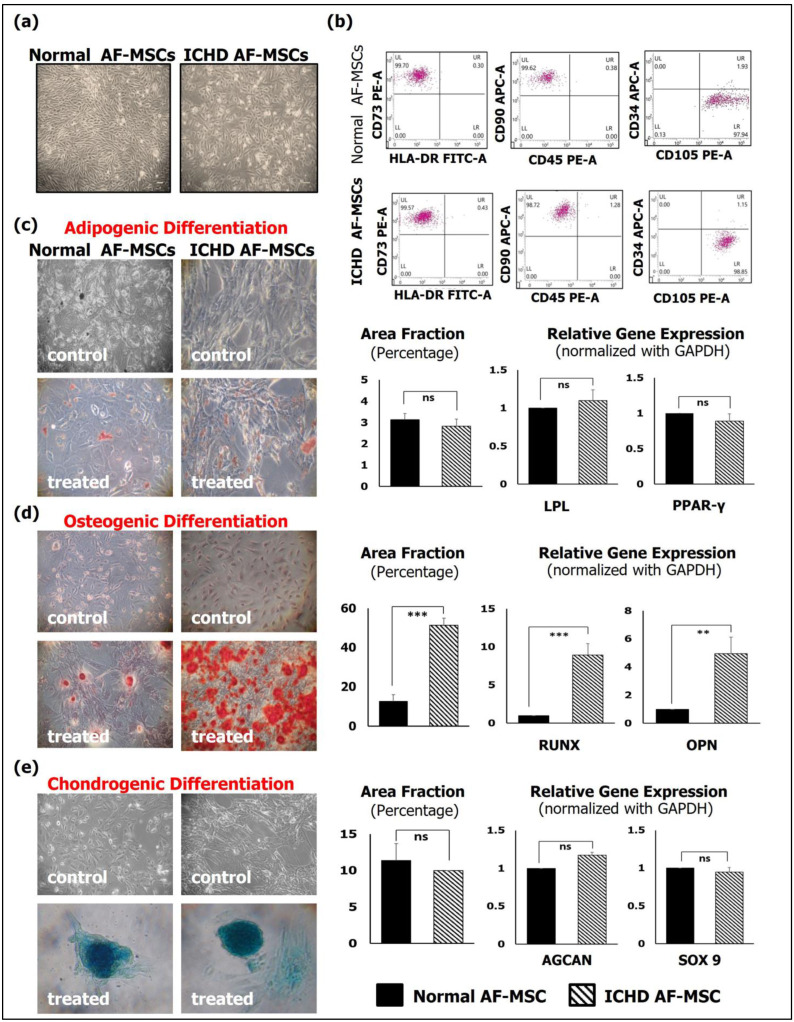
Morphology and characterization of normal and ICHD AF-MSCs. (**a**) Representative microphotographs of both types of AF-MSCs, showing uniform spindle-shaped morphology at P3 (10×, 10 μm). (**b**) Flow-cytometry dot plots of both normal and ICHD AF-MSCs, showing positive expression for MSC markers CD73, CD90, and CD105 and negative expression for HSC-specific markers HLA-DR, CD45, and CD34. (**c**) Representative photomicrographs of Oil Red O staining of normal and ICHD AF-MSCs on day 21 of incubation with adipogenic differentiation medium. The positively stained area was quantified using ImageJ and expression of adipogenic genes viz. *LPL* and *PPAR by qRT-PCR.* (**d**) Representative photomicrographs of Alizarin Red staining of normal and ICHD AF-MSCs on day 21 of incubation with osteogenic differentiation medium. The positively stained area was quantified using ImageJ and expression of osteogenic genes viz. RUNX and *OPN* by *qRT-PCR.* (**e**) Representative photomicrographs of Alcian Blue staining of normal and ICHD AF-MSCs on day 21 of incubation with chondrogenic differentiation medium. The positively stained area was quantified using ImageJ and expression of chondrogenic gene viz. *SOX9* and AGCAN by *qRT-PCR.* Control cells (untreated cells) were negative for Oil Red O, Alizarin Red, and Alcian Blue staining. Values expressed as mean ± SD; ** *p* < 0.01, *** *p* < 0.001.

Normal AF-MSCs and ICHD AF-MSCs showed variable morphologies, comprising spindle-shaped, epithelioid, and fibroblast-like shapes at passage zero (P0). After passage one, AF-MSCs of both types exhibited a uniform, spindle-shaped morphology (Figure 1a). Immunophenotypic characterization by flow cytometry revealed that normal AF-MSCs and ICHD AF-MSCs showed a comparable expression for MSC markers: CD73 (97.74 ± 0.4% vs. 97.91 ± 1.2%), CD90 (94.90 ± 2.3% vs. 94.85 ± 0.3%), and CD105 (96.01 ± 1.3% vs. 98.56 ± 1.17%) and less than 5% expression for hematopoietic stem cell markers CD34 (2.18 ± 0.7% vs. 2.09 ± 0.2%), CD45 (1.39 ± 0.3% vs. 0.30 ± 0.07%), and HLA-DR (2.31 ± 0.4% vs. 1.44 ± 0.3%) (Figure 1b). Assessment of trilineage differentiation potential of both normal and ICHD AF-MSCs into adipogenic, osteogenic, and chondrogenic lineages revealed comparable adipogenic and chondrogenic differentiation in both cell types. Phase contrast microscopy revealed that both normal and ICHD AF-MSCs formed comparable lipid droplets and spheroids when stained with Oil Red O and Alcian Blue, respectively. This was also evident from comparable gene expression levels of adipogenic and chondrogenic genes viz. *LPL* (1 ± 0 vs. 1.1 ± 0.1), *PPAR-Y* (1 ± 0 vs. 0.89 ± 0.2), *SOX9* (1 ± 0 vs. 1.175 ± 0.03), and *AGCAN* (1 ± 0 vs. 0.94 ± 0.06), respectively (Figure 1c, e). However, we observed a significant increase in the osteogenic differentiation potential of ICHD AF-MSCs as confirmed by staining bright orange red with Alizarin Red (Figure 1d). This increase in osteogenic lineage differentiation was accompanied by an increased expression of osteogenic genes viz. Runt-related transcription factor (*RUNX)* (1 ± 0 vs. 4.95 ± 1.16; *p* < 0.001) and Osteopontin (*OPN*) (1 ± 0 vs. 8.95 ± 1.48; *p* < 0.001) genes (Figure 1d). Collectively, these results reveal that the ICHD AF-MSCs have similar cell morphology, comparable expression of mesenchymal markers, and bi-lineage differentiation potential towards adipogenic and chondrogenic lineages compared to normal AF-MSCs. However, in terms of osteogenic differentiation, ICHD-AF-MSCs showed altered osteogenesis compared to normal AF-MSCs.

### 3.2. Growth Kinetics Studies of Normal AF-MSCs and ICHD AF-MSCs

**Figure 2:**
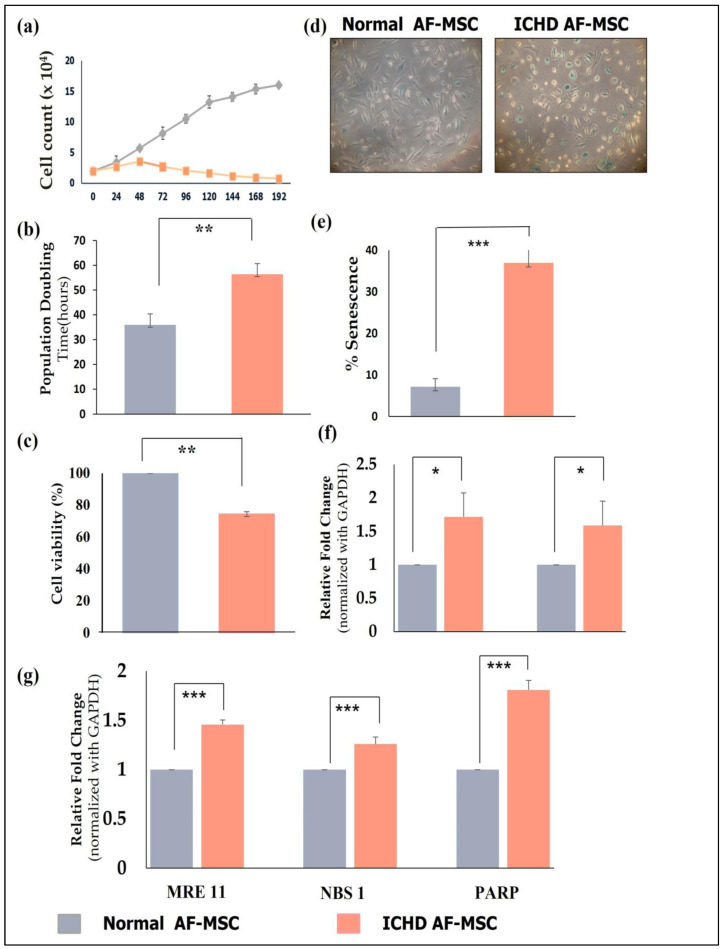
Proliferation, senescence, and DNA damage response in normal and ICHD AF-MSCs. (**a**) Growth curve of normal AF-MSCs and ICHD AF-MSCs at 24, 48, 72, 96, 120, 144, 168, and 192 h. (**b**) PDT (population doubling time) in passage 3, represented as a semi-logarithmic graph. (**c**) Percent cell viability in normal and ICHD AF-MSCs at P3. (**d**) Representative images of positively stained cells revealed by β-gal staining. (**e**) Quantification of percent senescent cells performed using ImageJ. (**f**) Relative gene expression of senescence-associated genes viz. TP53 and CDKN1A. (**g**) Relative gene expression of DNA damage response associated gene viz. MRE11, NBS 1 and PARP. Values expressed as mean ± SD; * *p*< 0.05, ** *p*< 0.01, *** *p* < 0.001.

Analysis of growth kinetics revealed that normal AF-MSCs showed a lag phase between 24 and 48 h, followed by an exponential phase until 192 h. In contrast, ICHD AF-MSCs showed an exponential phase up to 48 h, followed by a decline phase after 48 h (Figure 2a). Population doubling time (PDT) at passage 3 of normal AF-MSCs was also observed to be significantly lower than that of ICHD AF-MSCs (31.50 ± 0.6 vs. 56.56 ± 1.1; *p* < 0.01) (Figure 2b). Cell viability assays revealed significantly lower cell viability of ICHD-AFMSCs after 48 h of culture (100 ± 0% vs. 72.7 ± 0.019; *p* < 0.01) (Figure 2c). Senescence has been reported to be a contributing factor towards reduced cell proliferation and cell viability [23]; hence, we assessed senescence via β-galactosidase staining followed by the analysis of expression of senescence-associated genes by RT-PCR analysis in both AF-MSCs. We observed that in comparison to normal AF-MSCs, the ICHD AF-MSCs showed a significantly higher number of senescent cells (stained with blue) (7.19 ± 1.91% vs. 36.96 ± 5.08%; *p* < 0.001) along with elevated levels of senescence-associated genes viz. *TP53*(1 ± 0 vs. 1.71 ± 0.35; *p* < 0.05) and *CDKN1A* (1 ± 0 vs. 1.58 ±0.36; *p* < 0.01) (Figure 2d–f). Previous studies have reported the association of DNA damage with an increase in senescence; therefore, we checked if ICHD AF-MSCs have altered expression of DNA damage-response genes and found upregulated expression of *MRE11*(1 ± 0 vs. 1.45 ± 0.04; *p* < 0.01), *NBS1*(1 ± 0 vs. 1.25 ± 0.07; *p* < 0.05), and *PARP* (1 ± 0 vs. 1.8 ± 0.09 *p* < 0.01) genes in ICHD AF-MSCs (Figure 2g). Overall, these results revealed that ICDH AF-MSCs undergo increased senescence and DNA damage response.

### 3.3. Expression of Cardiac Progenitor Markers and Transcription Factors in Normal AF-MSCs and ICHD AF-MSCs

**Figure 3:**
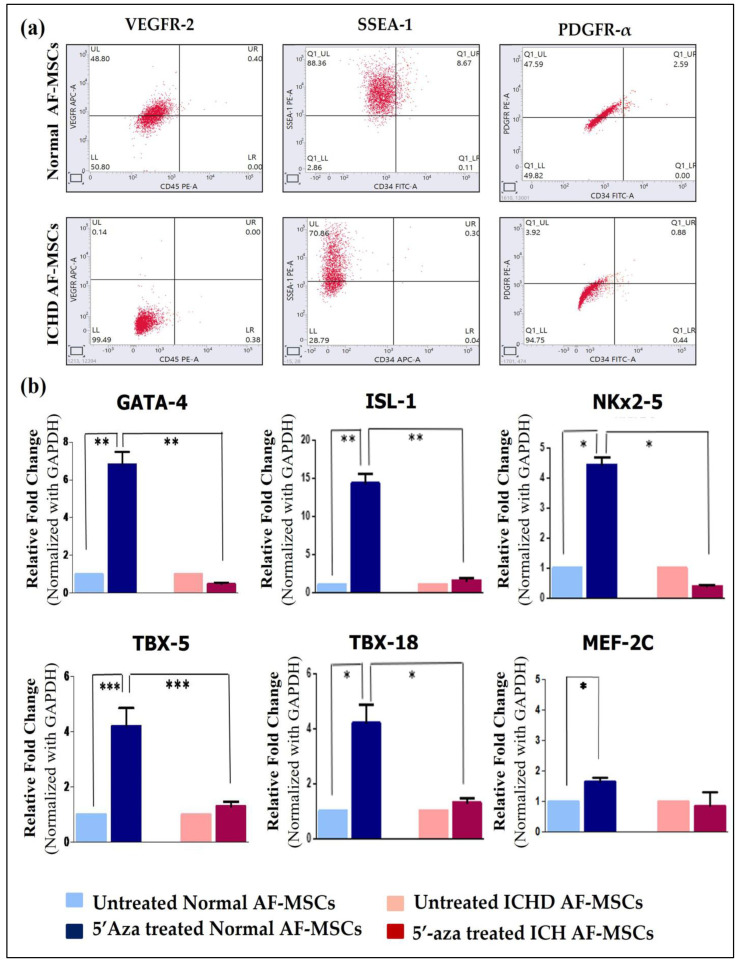
Expression of cardiac progenitor markers and cardiac transcription factors by normal AF-MSCs and ICHD AF-MSCs. (**a**) Representative flow cytometric dot plots of normal AF-MSCs and ICHD AF-MSCs expressing cardiac progenitor markers VEGFR-2, SSEA-1, and PDGFR-α (repetition = 03). (**b**) Real-time PCR showing expression of cardiac transcription factors viz. GATA-4, ISL-1, NKx 2-5, TBX-5, TBX-18, and MeF-2C in 5′-aza-treated normal AF-MSCs (dark blue bar) with 5′-aza-treated ICHD AF-MSCs (red bar). (1 × 10^6^ cells for each untreated and 5′-aza-treated ICHD and normal AF-MSCs). Values are expressed as mean ± SD; * *p* < 0.05, ** *p* < 0.01, *** *p* < 0.001.

Given the previous results confirming senescence and DNA damage in ICHD AF-MSCs, we wanted to investigate if these cells also have an altered cardiac differentiation potential compared to normal AF-MSCs. Therefore, we evaluated the expression of cardiac progenitor markers and cardiac transcription factors in these cells. Our findings confirmed that, compared to normal AF-MSCs, the ICHD-AF-MSCs showed a significant decrease in the expression of both cardiac progenitor markers VEGFR-2 (48.80 ± 0.9% vs. 0.14 ± 0.6%, *p* < 0.01), SSEA-1 (88.36 ± 2.7% vs. 70.86 ± 2.4%, *p* < 0.01), and PDGFR-α (47.59 ± 3.09% vs. 3.92 ± 1.8%, *p*< 0.01) (Figure 3a) and cardiac transcription factors *GATA-4* (6.8 ± 1.20 vs. 4 ± 0.1; *p* < 0.01), *ISL-1* (14.3 ± 1.12 vs. 2.3± 0.6; *p* < 0.01), *NKx 2-5* (14.1 ± 2.8 vs. 1.1 ± 0.3; *p* < 0.01), *TBX-5* (4.4 ± 0.3 vs. 0.4 ± 0.07; *p* < 0.001), *TBX-18* (4.19 ± 0.3 vs.1.3 ± 0.2; *p* < 0.01), and *MeF-2C* (1.9 ± 0.4 vs. 0.9 ± 0.05, *p* ≥ 0.05; ns) (Figure 3b). These results highlight differentiation defects in ICHD AF-MSCs.

### 3.4. Tri-Lineage Cardiovascular Differentiation Potential of Normal AF-MSCs and ICHD AF-MSCs

**Figure 4:**
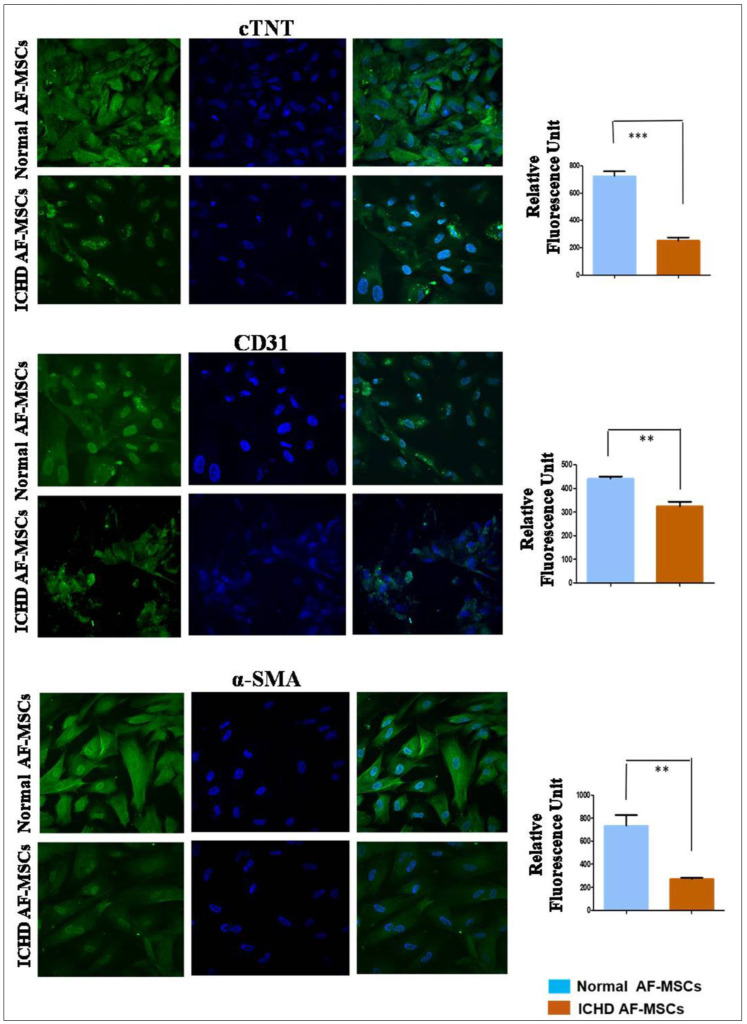
In vitro differentiation of normal and ICHD AF-MSCs into cardiomyocytic-like cells, endothelial cells, and alpha-smooth muscle actin. Representative immunofluorescence photomicrographs demonstrating the expression of cardiovascular marker proteins cTNT, CD31, and α-SMA in control and differentiated normal and ICHD AF-MSCs (5 × 10^4^ cells/well in 12-well plates for each ICHD and normal AF-MSC; scale bar, 20µm); quantification of fluorescence intensity for markers proteins cTNT, CD31, and α-SMA in differentiated normal and ICHD AF-MSCs. Values expressed as mean ± SD; ** *p* < 0.01, *** *p* < 0.001.

Normal AF-MSCs and ICHD AF-MSCs differentiate into cardiomyocytes, endothelial cells, and alpha-smooth muscle cells (as revealed by cTNT, CD31, and α-SMA expression, respectively). However, the expression of cTNT, CD31, and α-SMA was markedly lower in ICHD AF-MSCs than in normal AF-MSCs. The quantitative analysis of immunofluorescent images further confirmed that the expression level of these proteins in ICHD AF-MSCs was significantly lower in comparison to AF-MSCs viz. cTNT (*p* < 0.001), CD31 (*p* < 0.01), and α-SMA (*p* < 0.05), suggesting ICHD AF-MSCs have a poorer cardiovascular differentiation potential in comparison to the normal AF-MSCs (Figure 4). Taken together, these findings show that ICHD AF-MSCs have down-regulated expression of cardiac progenitor markers, transcription factors, and cardiovascular lineage-specific markers compared to normal AF-MSCs, thus highlighting tri-lineage cardiovascular differentiation defects in these cells.

## 4. Discussion

Our study demonstrates that AF-MSCs derived from ICHD fetuses exhibit a lower proliferation rate and increased senescence with an elevated DNA damage response compared to normal AF-MSCs. Further, it also highlights that ICHD AF-MSCs have defective cardiomyogenic differentiation potential as the expression of cardiac progenitor markers, cardiac transcription factors, and cardiovascular-specific markers were significantly lower in these cells compared to normal AF-MSCs. To the best of our knowledge, this is the first study demonstrating the proliferation and cardiomyogenic differentiation defects of AF-MSCs derived from ICHD fetuses in comparison to that of normal AF-MSCs.

We first compared ICHD AF-MSCs to normal AF-MSCs in terms of morphology, phenotype, and multi-potent differentiation potential. Upon culturing normal AF-MSCs and ICHD AF-MSCs to passage 3, both cell types exhibited uniform, spindle-shape morphology. These results regarding the morphology of AF-MSCs are consistent with previous reports by our and other groups [19,24]. Immunophenotypic characterization demonstrated that ICHD AF-MSCs showed positive expression for mesenchymal markers CD73, CD90, and CD105 and negative expression for the hematopoietic markers viz. CD34, CD45, and HLA-DR comparable to normal AF-MSCs. These results are in line with the previous studies on AF-MSCs [19]. In addition to the expression of cell surface markers, normal and ICHD AF MSCs showed the ability to differentiate into adipogenic, osteogenic, and chondrogenic lineages, which is one of the determining properties of MSCs [19,25]. In our study, we observed that the differentiation potential towards adipogenic and chondrogenic lineages was comparable in both normal and ICHD-AF MSCs as revealed by Oil Red O and Alcian Blue staining, respectively. This finding was further validated by the expression of genes associated with adipogenic and chondrogenic lineages. Interestingly, ICHD AF-MSCs showed significantly higher osteogenic differentiation potential than normal AF-MSCs, as shown by Alizarin Red staining. Osteogenic differentiation is tightly regulated by expression of osteogenic-specific transcription factor and osteogenic genes such as *RUNX* and *OPN,* respectively [26,27]; therefore, we assessed the expression of these genes. We observed that, in comparison to normal AF-MSCs, ICHD AF-MSCs exhibited an enhanced expression of these genes. The *RUNX* transcription factor (also called core binding factor A1) is the most essential for osteoblast commitment, differentiation, matrix production, and mineralization during bone formation, and it controls the regulation of osteogenic marker genes such as *OPN*. *OPN* is a non-collagenous protein that is secreted during the early and intermediate stages of osteogenesis [28,29]. Hence, up-regulation of these genes results in the increased production of a mineralized matrix in AF-MSCs derived from ICHD fetuses.

AF-MSCs derived from healthy fetuses are known to have a high proliferation capacity and are less prone to senescence [30,31,32,33]. However, in our study, the AF-MSCs derived from ICHD fetuses showed a decreased proliferation rate in comparison to normal AF-MSCs. At passage 3, we observed a significantly increased number of *β-galactosidase*-positive cells in ICHD AF-MSCs, suggesting increased senescence in these cells compared to normal AF-MSCs. It has been reported that induction of senescence in the cells can decrease cell proliferation [23,34,35]. Corroborating with these studies, we observed that a decrease in proliferation rate of ICHD-MSCs was accompanied by enhanced senescence. One pronounced feature of senescent cells is the loss of DNA damage-repair mechanisms [36]. Although senescence is associated with aging, cells can undergo senescence irrespective of age due to DNA damage, telomere shortening, and other senescence stimuli [37]. In line with this, we observed an increased expression of the DNA damage genes *MRE11*, *NBS1*, and *PARP* in ICHD AF-MSCs, suggesting impaired DNA repair in these cells compared to normal AF-MSCs.

It has been demonstrated that AF-MSCs express cardiac progenitor markers including PDGFR-α, VEGFR-2, and SSEA-1 [38]. Consistent with this, we observed that both normal and ICHD AF-MSCs expressed these cardiac progenitor markers. However, the expression of these markers was significantly lower in ICHD AF-MSCs, thus highlighting a defect at the progenitor stage in these stem cells. Cardiac progenitor markers expressing stem cells are resident populations involved in cardiac homeostasis and mediate the differentiation of partially differentiated cardiomyocytes into fully matured cardiomyocytes and, hence, maintain a functional heart. These markers are also involved in heart tube expansion into the anterior and ventricular regions [39]. PDGFR-α is also a mesenchymal marker, and PDGFR-α-positive cells can aid in vasculogenesis, heart tube assembly, and the formation of cardiac fibroblasts [40]. VEGFR-2 has been shown to play an essential role in angiogenesis and the development of heart valves via VEGFR/KDR signaling [41,42,43]. SSEA-1 is the most primitive mesenchymal progenitor marker. Several studies have shown that SSEA-1-enriched cells give rise to the left and right ventricles and play a role in cardiomyogenic differentiation [44] Thus, any defects in these cardiac progenitor factors can lead to defective cardiomyogenesis like in ICHD.

AF-MSCs can be differentiated into the cardiomyogenic lineage upon induction with the DNA methylation-inhibiting agent 5′-azacytidine, and the differentiation process is regulated by the expression of cardiac transcription factors like *GATA-4*, *ISL-1*, *NKx2-5*, *TBX-5*, *TBX-18*, and *MeF-2C* [19]. Consistent with this study, we observed that 5′-aza-treated normal AF-MSCs showed a significantly high fold change expression of transcripts for *GATA-4*, *ISL-1*, *NKx2-5*, *TBX-5*, *TBX-18*, and *MeF-2C* transcription factors. However, these transcription factors were found to be down-regulated in 5′-aza-treated ICHD AF-MSC, highlighting a defective cardiomyogenic differentiation process in these cells. These core cardiac transcription factors have been reported to interact with one another and with a diverse array of other transcription factors to control heart development. Many of the transcription factors are later repurposed to control cardiac chamber maturation, conduction system development, and endocardial cushion remodeling [45,46,47,48]. Therefore, it is not surprising that any alterations or mutations in these factors can lead to congenital heart disease. It has been reported that mutations in *GATA-4* and *NKX2-5* disrupted *TBX-5* recruitment, particularly to cardiac super-enhancers, and were concomitant with dysregulation of genes related to phenotypic abnormalities, including cardiac septation and cardiac functions [49,50,51]. Thus, one of the possibilities for the almost absent expression of these cardiac transcription factors in ICHD AF-MSCs may be genetic and epigenetic alterations in these cells, as previously described. However, further studies are warranted to confirm if the genetic or epigenetic alterations of the aforementioned factors contribute to the defective cardiomyogenic differentiation process in ICHD AF-MSCs.

In addition to cardiac transcription factors, we examined the expression of structural proteins such as cardiac troponin (cTNT), endothelial (CD31), and smooth muscle cells (SMA) in normal as well as ICHD AF-MSCs. Our findings revealed that both normal AF-MSCs and ICHD AF-MSCs differentiated into cardiovascular tri-lineages and expressed cTNT, CD31, and SMA; however, the expression of these proteins was significantly down-regulated in ICHD AF-MSCs compared to normal AF-MSCs, suggesting that ICHD AF-MSCs have lower efficiency of differentiating into cardiovascular lineages. Cardiac structural protein cTNT is a cytoplasmic protein known to function in cardiac muscles and play an important role as a part of the troponin complex of myofibrils. Smooth muscle actin (SMA) is a protein that regulates fibroblasts in mature myofibroblasts [52,53]. CD31 also plays a role in vasculogenesis in addition to being an endothelial marker [54]. Hence, alterations in the expression of these structural proteins might contribute to impairment in heart development during embryogenesis, as observed in the case of ICHDs. 

The present study reveals interesting findings about functional defects in AF-MSCs derived from ICHD fetuses; however, the study has some limitations. The number of samples taken in each group was quite small; hence, further studies with larger sample sizes are desired to consolidate the findings of this study. In addition, the molecular mechanism(s) behind these defects has not been explored. Thus, working toward an understanding of the mechanistic basis of these defects will facilitate our understanding of the pathobiology of ICHD in fetuses.

## 5. Conclusions

Overall, our study highlights that ICHD AF-MSCs have increased osteogenic differentiation potential and a lower proliferation rate along with elevated senescence and DNA damage response. Moreover, ICHD AF-MSCs expressed markedly lower levels of cardiac progenitor markers, cardiac-specific transcription factors, and cardiovascular-lineage-specific structural proteins, thus highlighting severe cardiomyogenic differentiation defects in these stem cells. The improper heart development in ICHD has been linked to genetic mutations, variations in single nucleotide polymorphisms (SNPs) and chromosomal copy number variants (CNVs) as well as alterations in the expression of genes associated with fetal heart development. Therefore, to explore if the ICHD AF-MSCs carry out these alterations, we plan to use next generation sequencing approaches, including whole genome sequencing (WGS) and RNA sequencing (RNA Seq.) WGS will help us to identify the molecular variants and identify frequent, rare, or novel co-occurring genetic aberrations contributing to the defects in ICHD-MSCs, which will help us understand the genetic variability of the disease. With RNA sequencing, we expect to identify differentially expressed genes associated with the decreased proliferation and altered cardiomyogenic differentiation potential of ICHD-AFMSCs. Overall, these results will provide new insight into the molecular mechanisms contributing to multiple defects in ICHD AF-MSCs, and this could lead to the identification of novel targets for ICHD diagnosis and prognosis.

## Figures and Tables

**Table 1:** List of antibodies with their dilutions.

Antibodies	Catalogue Number	Dilution	Source
CD73 (PE)	344,003	1:100	BioLegend, San Diego, CA, USA
CD90 (APC)	328,113	1:100	BioLegend, San Diego, CA, USA
CD105 (FITC)	323,203	1:100	BioLegend, San Diego, CA, USA
CD34 (FITC)	343,603	1:100	BioLegend, San Diego, CA, USA
CD45 (PE)	304,007	1:100	BioLegend, San Diego, CA, USA
HLA-DR (APC)	307,605	1:100	BioLegend, San Diego, CA, USA
PDGFR-α (PE)	323,505	1:100	BioLegend, San Diego, CA, USA
SSEA-1 (PE)	330,405	1:100	BioLegend, San Diego, CA, USA
VEGFR-2 (APC)	359,915	1:100	BioLegend, San Diego, CA, USA
cTNT	ab45932	1:200	Abcam, Cambridge, UK
anti-rabbit secondary antibodies (FITC)	ab6717	1:200	Abcam, Cambridge, UK
CD31	ab24590	1:100	Abcam, Cambridge, UK
α-SMA	ab5694	1: 50	Abcam, Cambridge, UK

**Table 2:** Primer sequences of the target genes.

Gene Name	Target Gene-Primer Sequence
*Lipoprotein Lipase*	Forward-5′ TCCAAACCAGAAAACGGAAG3′ Reverse-5′ ACAGCCAGTCCACCACAATG3′
*PPAR-ϒ*	Forward-5′ TCAGGGCTGCCAGTTTCG 3′ Reverse-5′GCTTTTGGCATACTCTGTGATCTC 3′
*Osteopontin (OPN)*	Forward-5′ CCTGCCAGCAACCGAAGT 3′ Reverse-5′ CCTCGGCCATCATATGTGTCT 3′
*RUNX*	Forward-5′ TCGAATGGCAGCACGCTAT 3′ Reverse-5′ CATCAGCGTCAACACCATCAT 3′
*SOX 9*	Forward-5′ AGCGACGTCATCTCCAACATC 3′ Reverse-5′ GTTGGGCGGCAGGTACTG 3′
*AGCAN*	Forward-5′ GGAAGGCTGCTATGGAGACAAG 3′ Reverse-5′ GGTGTCTCGGATGCCATACG 3′
*GATA-4*	Forward-5′TCCAAACCAGAAAACGGAAG3′ Reverse-5′CTGTGCCCGTAGTGAGATGA3′
*NKx 2-5*	Forward -5′AGTTTGTGGCGGCGATTAT3′ Reverse-5′AGCTCAGTCCCAGTTCCA3′
*ISL-1*	Forward-5′GCCTTGCAGAGTGACATAGAT3′ Reverse-5′CTGGAAGTTGAGAGGACATTGA3′
*TBX-5*	Forward-5′AACCACAAGATCACGCAATTAAAG3′ Reverse-5′GTCATCACTGCCCCGAAATC3′
*TBX-18*	Forward-5′CGGTGGAGGCGCTGATC3′ Reverse-5′CAGTTTTCGCCGCTTCT3′
*MeF-2C*	Forward-5′CACCAGGCAGCAAGAATACGA3′ Reverse-5′CTCAGCCGACTGGGAGTTATTT3′
*TP53*	Forward-5′GTCCCAAGCAATGGATGATTTG3′ Reverse-5′GCATTCTGGGAGCTTCATCT3′
*CDKN1A*	Forward -5′TGGAGACTCTCAGGGTCGAAAA3′ Reverse-5′CGGCGTTTGGAGTGGTAGAA3′
*MRE11*	Forward-5′GTGGACAAGGAGGAGAAAGATG3′ Reverse-5′TGTCTTCGAGGGCATCAATATG3′
*NBS1*	Forward-5′GTCAGGACGGCAGGAAAGAA3′ Reverse-5′TCAACCTAGCTTCCCCACCT3′
*PARP*	Forward-5′AGTGCCAACTACTGCCATAC3′ Reverse-5′AGCGTGCTTCAGTTCATACA3′

## Data Availability

Not applicable.
